# Coupling of Anodic Oxidation and Soil Remediation Processes: A Review

**DOI:** 10.3390/ma13194309

**Published:** 2020-09-27

**Authors:** Maiara Barbosa Ferreira, Aline Maria Sales Solano, Elisama Vieira dos Santos, Carlos A. Martínez-Huitle, Soliu O. Ganiyu

**Affiliations:** 1 Instituto de Química, Universidade Federal do Rio Grande do Norte, Natal 59078-970, RN, Brazil; mahbarbosaferreira@hotmail.com (M.B.F.); lininhamss@hotmail.com (A.M.S.S.); elisama_quimica@yahoo.com.br (E.V.d.S.); 2 Department of Civil and Environmental Engineering, University of Alberta, Edmonton, AB T6G 2W2, Canada

**Keywords:** anodic oxidation, soil remediation, surfactants, biosurfactants, microemulsion, electrokinetic

## Abstract

In recent years, due to industrial modernization and agricultural mechanization, several environmental consequences have been observed, which make sustainable development difficult. Soil, as an important component of ecosystem and a key resource for the survival of human and animals, has been under constant contamination from different human activities. Contaminated soils and sites require remediation not only because of the hazardous threat it possess to the environment but also due to the shortage of fresh land for both agriculture and urbanization. Combined or coupled remediation technologies are one of the efficient processes for the treatment of contaminated soils. In these technologies, two or more soil remediation techniques are applied simultaneously or sequentially, in which one technique complements the other, making the treatment very efficient. Coupling anodic oxidation (AO) and soil remediation for the treatment of soil contaminated with organics has been studied via two configurations: (i) soil remediation, ex situ AO, where AO is used as a post-treatment stage for the treatment of effluents from soil remediation process and (ii) soil remediation, in situ AO, where both processes are applied simultaneously. The former is the most widely investigated configuration of the combined processes, while the latter is less common due to the greater diffusion dependency of AO as an electrode process. In this review, the concept of soil washing (SW)/soil flushing (SF) and electrokinetic as soil remediation techniques are briefly explained followed by a discussion of different configurations of combined AO and soil remediation.

## 1. Introduction

An industrial revolution and increased agricultural mechanization in the last decades have had several consequences, including environmental issues and sustainable development. Decision and policy makers in industries, economics, and politics take careful consideration of these topics and several stringent rules and regulations on environmental pollution issues are being promulgated annually, especially on hazardous waste, atmospheric pollution, and wastewater [1,2]. Soil, an important component of ecosystem and a key resources for the survival of human and animals, has been under constant contamination from different human activities such as excessive use of pesticides and fertilizer on farmlands, infiltration from livestock impoundments, accidental discharge of harmful pollutants, industrial wastewater, landfill leachates, petroleum spillage and oil rigging, as well as improper waste disposal and stockpiles, all of which constitutes serious soil pollution and deterioration of soil quality [3,4]. As such, soil contamination is a global issue and is considered to be one of the barriers for sustainable development. The major soil contaminants include heavy metals and toxic anions, toxic organic compounds, and radionuclide [5]. Heavy metal contaminants such as Cr, Hg, Cd, and Pb, as well as organic pollutants like volatile chlorinate solvent, polycyclic aromatic hydrocarbons (PAHs), polychlorinated biphenyls (PCBs), and total petroleum hydrocarbons (TPHs) have been reportedly found at different contamination levels in soils across the globe [6,7,8]. These contaminants pose a great threat to the safety of the ecosystem and human health via food chain and direct exposure to the contaminated soils. Additionally, organic contaminants hardly degrade in soil by abiotic/biotic agents, and thus persist and accumulate [2,3].

Due to their hazardous and potential risk to human health and safety of the ecosystem, as well as land reuse, contaminated soils require effective remediation for their reclamation and reuse. In-depth works have been devoted to the development of soil remediation techniques, and several new and innovative solutions for effective abatement of contaminants from soils have been investigated either to completely eliminate the contaminants or reduce their concentrations to tolerable and safe levels [3,9,10]. Most soil remediation processes that are in existence have at least one major “bottle-neck”, such as high cost (thermal treatment), low efficiency (pump and treat), long treatment time (biological treatment), low treatment capacity and high chemical requirement (in situ chemical oxidation), or necessity for post-treatment decontamination of the effluents (soil washing (SW)/soil flushing (SF) systems) [2,11]. Sequential or combined remediation techniques in which two or more treatment techniques are applied either simultaneously or sequentially have the advantage of each technique complementing the merits and overcoming the challenges of each other [1]. Among the existing combined techniques, SW/SF/electrokinetic soil remediation coupled with advanced oxidation processes (AOPs) is a versatile and time-efficient treatment for remediation organic pollutant contaminated soils and it has been used globally over the years [2,12]. SW is a mechanical process, which involves the use of liquids (extracting agents) to remove chemical pollutants from the soils [2,6,13,14]. However, the contaminants are only separated into solution, which implies that necessary treatment is still required for the washing effluents before discharge into the environment. AOPs based on hydroxyl radical productions (^•^OH) such as ozonation, photocatalysis, Fenton oxidation, and electrochemical AOPs have been recently investigated for treating SW effluents with the best removal efficiency observed with electrochemical AOPs [2,3,12,15,16,17]. Among the electrochemical AOPs, electrooxidation otherwise known as anodic oxidation (AO), has a distinguished advantage in that it requires very limited or no chemical for the generation of strong oxidant (reactive species) needed for oxidation of the organic pollutants [18,19,20,21]. In AO, reactive oxygen species (ROS) or reactive chloride species (RCS) are electrogenerated at the anode region via water/chlorine oxidation [22,23,24,25]. The produced reactive oxygen species, especially ^•^OH, is a very strong oxidant (E = −2.8 eV vs. SHE) and it can react non-selectively with any class of organic pollutants until their total conversion to CO_2_ or at least achieve high mineralization of the organics [26,27,28,29]. The fundamental principles and application of this process are available in literatures [30,31,32,33,34].

**Figure 1:**
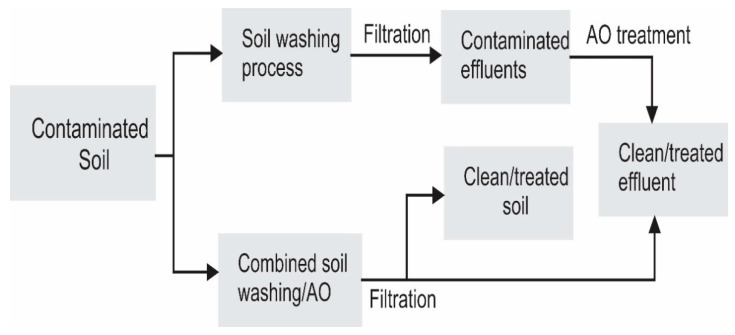
Schematic of different configurations of coupling soil washing (SW) with anodic oxidation (AO).

Coupling of AO and soil remediation for the treatment of organic contaminated soils has been studied via the following two configurations (Figure 1): (i) AO as a post-treatment stage for the treatment of SW/SF/electrokinetic soil remediation effluents and (ii) in situ and simultaneous AO and SW/SF process. The former is the most widely investigated configuration of the combined soil remediation and AO processes, whereas the latter is less common, owing to the higher diffusion dependency of the electrode process-like AO. In this review, the concept of SW/SF/electrokinetic soil remediation is briefly explained followed by a discussion of different configuration of combined AO and soil remediation. In the final section, we provide a brief future perspective and concluding remarks.

## 2. Soil Remediation Processes

Remediation of contaminated soils has become a global issue due to the hazardous effect of contaminants on the ecosystem and also due to land shortages that necessitate reclamation of contaminated sites for reuse. Among the existing soil remediation techniques, SW/SF is a versatile, cost- and time-efficient method and has attracted increasing attentions in recent years across the globe [2,6,13]. Another rising and recently widely studied technique is the electrokinetic soil remediation process in which electric field produced by low DC current applied across the soil surface separates the contaminants from soil with the aid of added electrolytes [12,35]. Unfortunately, both processes generate effluents which contain the pollutants extracted from the soil and require extensive and proper treatment before disposal.

### 2.1. SW/SF Processes

**Figure 2:**
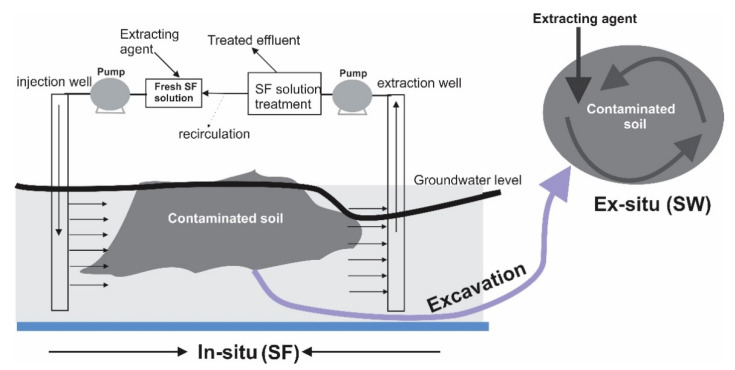
Scheme of a typical soil flushing and SW processes. Adapted from [2].

In SW/SF, extracting agents, usually in aqueous solutions are used to mechanically remove chemical pollutants from contaminated soils [6,12]. Contaminants usually have low solubility and adhere strongly to the surface of the soil in real contaminated sites, as such, in practical SW processes, additives such as acids, surfactants, and chelating agents are often added to washing solution to solubilize the contaminants from the soil [6]. SF is an in situ process where extracting agents are added to the contaminated soils to improve the mobility of pollutants by reducing the interfacial tension between them and the groundwater [2,12,36]. Then, the mobilized pollutants can be displaced in the extraction wells (Figure 2). SF is more adapted to light-weight polycyclic aromatic hydrocarbons (PAHs) like naphthalene remediation because the pumping can be easily operated from the surface of the groundwater table [2]. The contaminated site and field characteristics such as soil heterogeneity, contaminant nature, naphthalene saturation, and others, strongly influence the efficiency of the SF process [37]. SW is an ex situ process (Figure 2) where the contaminated soil and site are excavated, transported, and treated with a certain soil/liquid ratio, usually between 5–45% [14,38]. The contaminants sorbed to the soil are removed by adding extracting agents to the washing solution and there is always enhanced contact between the extracting agents and the soil contaminants in SW, thereby allowing better treatment efficiency assessment as compared with the SF process [14].

### 2.2. Extracting Agents

**Figure 3:**
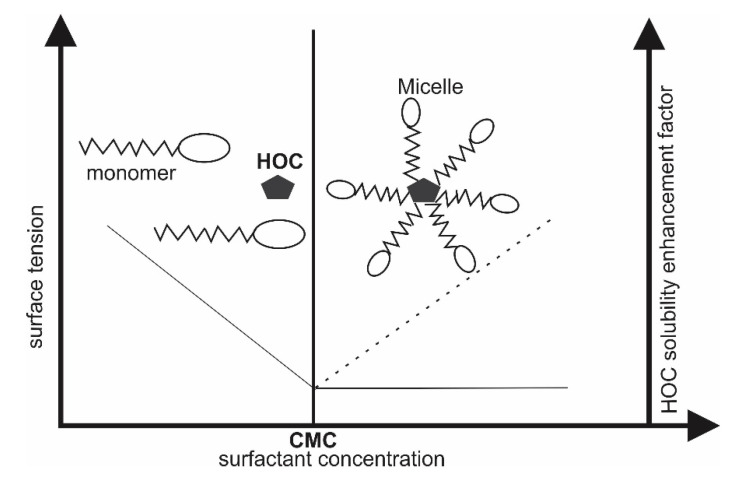
Surface tension and hydrophobic organic compound (HOC) solubility enhancement factor as a function of surfactant concentration. Printed with the permission of [2].

**Figure 4:**
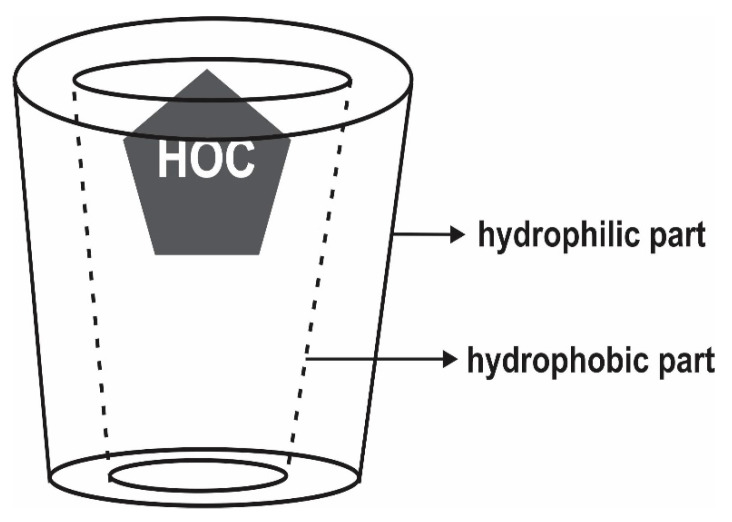
Inclusion complex formed by cyclodextrins (CDs) with hydrophobic organic compounds (HOCs) during SW. Reprinted with the permission of [2].

The aqueous solutions with or without additives used to mobilize the contaminants from the soil to the SW solution are termed extracting agents. In addition to having good extracting and solubilizing ability, the extracting agents should possess excellent biodegradability and low eco-toxicity to the soil organisms, as well as environmental compartments where it is disposed after usage [36]. Extracting agents can also mobilize non-targeted contaminants especially heavy metals and toxic anions such as lead, cadmium, chromium, copper, and arsenic, and thus the SW effluents require proper treatments and toxicity assessment before disposal [2,6]. Some of the commonly used extracting agents are briefly described as follows:

- **Water and organic solvents:** Organic solvents are the earliest extracting agents used for the removal of PAHs from contaminated soils, both on bench and field scales [13,39]. Hydrophobic soil contaminants such as PAHs with lower octanol-water partition coefficient (log K_OW_) have been removed from contaminated soils using non-polar organic solvents including 1-pentanol, n-hexane, benzene, toluene, and dichloromethane [39]. To date, water and several organic solvents including alcohols, esters, ketones, alkylamines, and aromatics have been studied to extract PAHs from soils, however, organic solvents such as ethanol, 2-propanol, 1-pentanol, and ethyl acetate have been reported to be only effective for the removal of lower molecular weight (LMW) PAHs and less efficiency for higher molecular weight (HMW) PAHs, which continue to persist in soil after extraction [40,41].
- **Synthetic surfactants:** Surfactants are a class of amphiphilic chemicals composed of hydrophilic water-soluble heads and hydrophobic or water insoluble tails. Their unique molecular structure give them the ability to solubilize relatively insoluble xenobiotics including hydrophobic soil contaminants [6]. They are characterized by their chemical structures, hydrophobic-lipophobic balance, and their critical micellar concentration (CMC). The latter is defined as the surfactant concentration above which micelles are formed and all additional surfactants added to the solution go to the micelles (Figure 3) [36,42,43]. The solubility of hydrophobic organic compounds (HOCs) is strongly enhanced at a surfactant concentration above the CMC along with a decrease in surface tension [36]. Three different mechanisms are reported to be involved in surfactant enhanced removal of hydrophobic organics sorbed to soils, which include a decrease in interfacial tension, phase transfer of HOCs from soil-liquid interface to micellar pseudo-aqueous phase, and solubilization of the HOCs inside the hydrophobic enclosure formed by micelles [44]. Surfactants can be classified as anionic (e.g., sodium dodecylsulfate and linear alkylbenzene sulfonate), cationic (quaternary ammonium derivatives), non-ionic (Brij 35, Tween 80, Triton X-100), and amphoteric (cocoamidopropyl hydroxylsultaine), but non-ionic surfactants are preferred due to their lower soil sorption ability, cost effectiveness, and higher solubilization capacity [36]. Several studies have reported the use of different synthetic surfactants for the remediation of PAHs from contaminated soils both on laboratory and pilot scales [2,6,12,13,14].
- **Biosurfactants:** These are amphiphilic chemicals similar to synthetic surfactant but having microbial origin. They are capable of forming micelles and are manufactured from renewable resources such as water-soluble carbon sources, water-immiscible substrates, and nitrogen sources [45]. Biosurfactants have some distinguished advantages such as high extraction efficiency, extremely biodegradable, ecological safety, lower toxicity, and the possibility of produced in situ during the SW process [13]. Indeed, Lai et al. (2009) [46] reported higher petroleum hydrocarbon removal efficiency from contaminated soil with biosurfactants Rhamnolipid and Saponin as compared twithsynthetic surfactants Tween 80 and TX100 during the SW process. However, the ability to produce sufficient quantities during the SW/SF process at an economical rate is a major challenge of using biosurfactants.
- **Microemulsion:** These are optically transparent and thermodynamically stable single phase, usually prepared from a ternary mixture of water, water-immiscible oil, and a cosurfactant [13,47]. Water-based microemulsions behave like a separate bulk phase which is capable of desorbing and concentrating pollutants from soil [48]. Unlike synthetic surfactants where the extent of solubility enhancement sharply increases at the CMC, the extent of solubility enhancement is linearly proportional to the concentration of microemulsions [13]. Sodium castor oil sulfate, fatty ester water non-ionic surfactants (methyl ester from babassus oil and unsaturated fraction of palm oil), 1-butanol oil, and other microemulsions based on vegetable oil have been demonstrated to show higher extraction efficiency for several organic pollutants, especially PAHs and TPHs [47,48,49].
- **Cyclodextrins (CDs**)**:** CDs have been proposed as a non-toxic and highly biodegradable alternative to organic solvents and surfactants due to environmental concerns associated with removing PAHs from contaminated soils [50,51]. They consist of hydrophilic groups on the external side of their ring, which can dissolve in water and a low-polarity cavity providing hydrophobic matrix that can entrap many organic compounds into the rings (Figure 4). This characteristic provides CDs with a larger capacity in solubilizing hydrophobic contaminants such as PAHs [50,52,53]. CDs such as β-cyclodextrins, hydroxylpropyl-β-cyclodextrins, methyl-β-cyclodextrins, and recently chemically modified CDs have been investigated by batch experiments for desorption of PAHs from soils [50,54,55,56,57].
- **Humic acids (HAs)** HAs are the fraction of humic substance compose in soil that is insoluble in water under acidic conditions [13]. Conte et al. [58] hypothesized, for first time, that HAs were capable of reducing sorption of organic contaminants onto the soil. Subsequently, the same authors [59] reported the removal of PAHs from soil using HAs as natural surfactants. HAs, as extracting agents, showed similar extraction efficiencies to that of synthetic surfactants and achieved more than 80% PAHs removal from the contaminated soil. Some studies have utilized low molecular weight organic acids mainly released by plants [60] and soil nanoparticles which composed mainly organic contents (along with some inorganic clays) [61] to absorb organic compounds from contaminated soils and enhanced soil water solubility during the subsequent SW process.
- **Vegetable oils and organic cosolvents:** Due to several drawbacks such as high cost, risk of handling and storing, toxicity, and soil permeability disturbance, organic co-solvents are no longer considered to be promising extracting agents for the soil remediation process. Vegetable oils, which are composed of high triglycerides and have high affinity for PAHs, are favorable alternative/replacement to costly, toxic and non-biodegradable solvents and surfactants for removing PAHs from soil [13,62]. The planner aromatic rings of PAH molecules bind to the triglycerides structure of the vegetable oils during the SW. This is possible because vegetable oils are characterized by their hydrophobicity and long aliphatic carbon chain structure which forms hydrophobic interaction with non-polar molecules [13]. The use of vegetable oils for removing PAHs from contaminated soils has also been reported in the literature [63,64,65,66].

### 2.3. Electrokinetic Soil Remediation (EKSR)

Similar to SW/SF processes, electrokinetic soil remediation (EKSR) desorbs pollutants from contaminated soils and produces contaminant loaded effluents that require proper treatment before disposal. In EKSR, contaminants are desorbed from the soil by the electric field created within the contaminated soil by the application of direct current via electrodes located at the soil subsurface [12,67,68]. In addition to electrokinetic phenomena such as electro-osmosis, electromigration, electropherensis, etc., the applied current simultaneously initiates many physical phenomena (heating, change in viscosity, etc.), electrochemical reactions (water oxidation and reduction, H_2_ evolution), and chemical processes (ion exchange, dissolution of precipitates, etc.) which significantly change the soil [12]. These processes can be systematically combined by setting optimum configurations and operation conditions in a soil treatment process which can promote certain approaches such as electrochemical soil flushing to ensure the removal of many inorganic and organic contaminants from the soil and minimize other non-beneficial phenomena or reactions (such as heating, H_2_ evolution, etc.) [69,70,71,72,73]. This technology has been investigated for the remediation of different type of contaminated soils and, in particular, has been more effective for fine-grained soils with low hydraulic conductivities and large specific surface areas [68]. The main phenomenon during the treatment of such fine-grained soil is electro-osmosis, which involves accumulation of net electric charge at the surface of the solids in contact with the electrolyte solution and the accumulation of a thin counterion layer (electrical double layer/Debye layer) of the liquid surrounding the solid surface. Since the Debye layer is charged, this portion of the fluid is mobile within the electric field between the electrodes due to attraction and repulsion from the opposite and the same charge, respectively [12].

Electrokinetic soil flushing involves the driving of the ground water and added aqueous solutions (chemicals) in the soil to mobilize the pollutants in the soil. The pollutants are washed out of the soil with the aid of water/solution via dissolution of precipitates, ionic exchange, desorption, or by simple mechanic dragging during washing, thus, the pollutants are transferred from the soil into the water/solution, solving the soil contamination issues. The process is only economical for treating low permeability soils with small hydraulic flux where conventional SF is ineffective. However, for highly permeable soils, conventional SF driving by pressure gradients is sufficient in order to avoid the cost/expenses associated with the application of electrochemical technologies [12]. In a typical EKSR, the electro-osmotic flux mobilizes groundwater from anode to cathode and the water at the cathode can be recycled by pumping it into the anode to begin a new flushing process [74]. Electrode materials such as platinum, graphite, platinized titanium, carbon felt, stainless steel, and platinum-coated graphite have been applied as either anode or cathode in electrokinetic flushing process, however, the configuration of the electrodes depends on the reactor designs and the nature of the contamination in soil [12].

Several flushing fluids have been used with or without additives to mobilize pollutants in contaminated soils. Among them, the fluids that are capable of soil pH regulation are more beneficial because they can compensate for the influence of the acidic or alkaline fronts created in the soil during electrokinetic remediation [12,75,76]. These fluids can be use alone or along with surfactants such as Tween 80, SDS, β-CD, and others for the soil flushing. A buffer solution of Na_2_CO_3_/NaHCO_3_ which neutralizes the acidic fronts and acetic acid which neutralizes the alkaline fronts and lowers the treated soil pH are the important reagents used in soil flushing [77,78]. Other fluids such as citric acid, NaNO_3_, and NaHPO_4_ have been studied either alone or with surfactants for the remediation of soil contaminated with organic pollutants [12,79,80,81].

As stated earlier, in the SW/SF and ESKR processes, the effluents are loaded with both the extracting agents and the extracted organic contaminants. Therefore, necessary treatments are required to remediate the organic pollutants to harmless or biodegradable substances prior to disposal to the environment. Note that, some studies have reported in situ remediation using peroxidation with H_2_O_2_ and Fenton oxidation (FeSO_4_ and H_2_O_2_) simultaneously along with electrokinetic SF for treating contaminated soil [82,83]. However, the effluents generated from such combined processes still contain significant quantities of extracted contaminants, which explain the inadequacy of such oxidation process for complete decontamination of electrokinetic SF effluents.

## 3. Anodic Oxidation: Basic Principle and Electrode Materials

### 3.1. Principle

Anodic oxidation (AO) also known as electrooxidation is one of the most widely applied electrochemical advanced oxidation processes (EAOPs) due to its excellent efficiency, limited chemical requirement, ease of operation, as well as being environmentally friendly. This process has been extensively applied for the remediation of different classes of organic pollutants from wastewater, landfill leachate, ground water, contaminated soils, reverse osmosis concentrate, and others [20,22,84]. The main reactive species in AO is either chemisorbed oxygen/superoxide or physisorbed hydroxyl radicals depending on the electrocatalyst material used as electrode. The first step in the generation of reactive species in AO is the water oxidation at the anode surface to ^•^OH radicals (Equation (1)).

M + H_2_O → M(^•^OH) + OH^−^ + e^−^ (1)

M(^•^OH) → MO + H^+^ (2)

The subsequent step depends on the nature of the electrocatalyst material used as the anode of the electrochemical reactor. Some materials allow further oxidation of the generated ^•^OH radicals to form chemisorbed oxygen or superoxide (Equation (2)), whereas other materials interact weakly with the ^•^OH radicals and allow it to freely react with the content of the electrolyzed solution. The former electrocatalyst materials are called “active” electrodes and can only achieve electrochemical conversion of organic pollutants with limited mineralization, while the latter are termed “non-active” electrode materials which are very efficient for both degradation and electrochemical combustion of different classes of organic pollutants [20,85,86]. Different electrode materials have been utilized as the anode in AO and a brief summary of these materials is presented in Section 3.2. Cathode materials or counter electrodes in AO have limited contribution to the degradation of organic pollutants except in a few situations where a weak oxidant such as hydrogen peroxide is generated, which aids the degradation of the organics [18]. It is important to state that other secondary reactive species such as sulfate, carbonate, and chlorine species can be generated which depends on the electrode material used, applied current density, and water matrix/composition [85,87].

### 3.2. Electrode Materials for the AO Process

An electrode or electrocatalyst materials used as electrode, especially an anode is a key factor that determines the efficiency, energy consumption, and cost of the AO treatment process. The conductivity of the electrocatalyst materials dictates the energy consumption and the activity of the materials determines the efficiency of the process. Therefore, selection of electrode material is a key issue in all electrochemical oxidation processes. Some of the electrocatalyst materials are inexpensive but they possess low electrocatalytic activity, whereas others have excellent electrocatalytic activity but are very expensive. Thus, there is need for careful selection of electrode materials based on desired efficiency, cost, and environmental consideration. The major electrode materials currently in use in the AO process are discussed in following sections.

#### 3.2.1. Carbonaceous Materials

Carbon and graphite-based electrode materials have been extensively used for the removal of organic pollutants in electrochemical reactors in three-dimensional electrodes such as fluidized bed, particulate and porous electrodes, as well as plate. They are very inexpensive with a large surface area, good electrical conductivity, and corrosion resistance but generally they are unstable when conducting AO at a higher potential due to severe corrosion that reduces their activity and service life [20]. Carbon materials such as carbon felt, graphite felt, carbon pallet, glassy carbon, graphite particle, activated carbon, and carbon nanotubes have been studied as suitable electrocatalyst in the AO process. In some cases, carbon-based electrocatalysts can combine adsorption with electrochemical degradation of pollutants, thus, enhanced the efficiency of the process [20,88]. The oxidation of organic pollutants by carbon-based electrocatalysts has been reported to be via direct electron transfer at an applied potential below the oxygen evolution potential or the oxygenated functional groups bonded to the surface of the carbon electrodes, which behave differently than those generated on metallic active and non-active anodes [20,89,90].

#### 3.2.2. Dimensionally Stable Anode Materials (DSAs)

**Figure 5:**
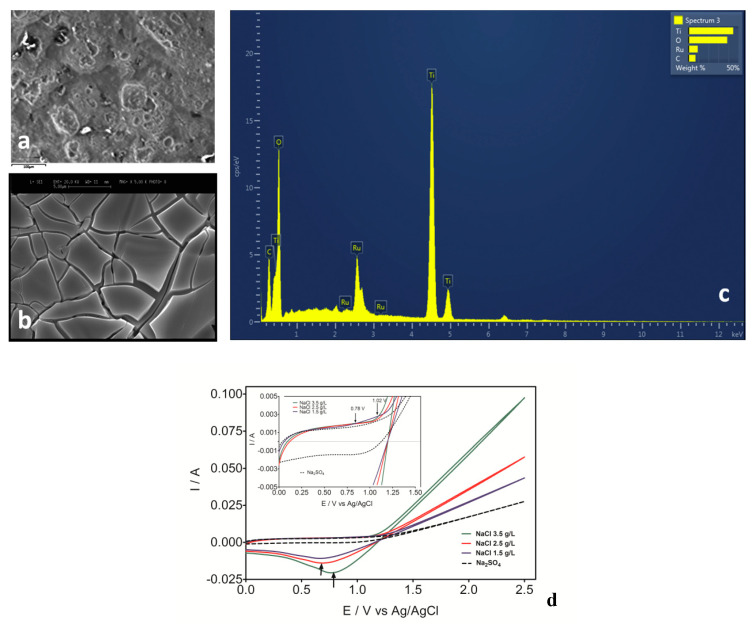
(**a**,**b**) SEM images; (**c**) EDS; (**d**) Linear scan voltammetry, of Ti/Ru_0.7_Ti_0.3_O_2_ electrode synthesized by thermal decomposition. Reprinted with the permission of [91].

Dimensionally stable anode materials (DSAs) belong to a class of electrocatalyst materials that consist of titanium-based metal substrate coated with a thin layer of conducting ruthenium, iridium, or thallium oxides. The conducting oxide layers can also be mixed metal oxides made from stoichiometry ruthenium, iridium, and thallium oxides. These electrodes are highly stable with respect to corrosive medium and high potentials and are good electrocatalysts for both chlorine and oxygen evolution [20,91,92]. Thin-film RuO_2_, Sb-Sn- RuO_2_, Ru_0.3_Ti_0.7_O_2_, RuO_2_-TiO_2_, IrO_2_, IrO_2_-RuO_2_, RuO_2_-IrO_2_-TiO_2,_ Ru_x_Ir_x−1_O_2_, IrO_2_-RuO_2_-SiO_2_, IrO_2_-Ta_2_O_5_, and others are some of the DSA electrodes that have been utilized in AO treatment of organic pollutants [20]. Many technologies, including sol-gel-thermal decomposition process, laser technology, electrodeposition, thermal decomposition, Pechini, and modified Pechini methods, have both been applied in laboratories and commercially for the preparation of DSA electrodes [20]. The synthesis technology has a strong influence on the physicochemical, chemical, and electrochemical properties, as well as the electrocatalytic activity of the DSA electrode. A typical physicochemical and electrochemical characteristic of Ti/Ru_0.3_Ti_0.7_O_2_ synthesized by thermal decomposition is shown in Figure 5. In AO with DSA electrodes, chemisorbed oxygen is the main oxidant generated in the anode region, except in chloride medium, where reactive chlorine species, which are relatively strong oxidants for the degradation of organic pollutants are generated in abundance [20,28,93].

#### 3.2.3. Platinum

Platinum electrodes have been extensively applied in the AO process for a long period of time due to their good conductivity and chemical stability, even at high potentials. These electrodes are classified as active anodes with low oxygen evolution overpotential (~1.6 V vs. SHE in 0.5 M H_2_SO_4_), and therefore behave in a similar manner as DSA with regards to organic pollutants oxidation [20,94,95]. Thin films deposited on suitable substrate, such as platinized niobium and bulk platinum (Pt) have both been utilized for the AO treatment of different classes of organic pollutants. The technologies for the production of Pt electrodes have matured and many companies, especially in European countries, are producing these electrodes commercially. Additionally, the many applications of Pt electrodes in different aspect of electrochemistry have aided the evolution of Pt electrode preparation technologies [20].

#### 3.2.4. Doped Lead Oxide (PbO_2_)

Pristine and doped lead oxide (PbO_2_) are excellent electrocatalyst materials for electrochemical oxidation of organic pollutants. They exhibit good conductivity, chemical stability, large surface area, and they are inexpensive and easy to prepare [20]. The development of PbO_2_ electrodes for electrochemical oxidation of organics has attracted great interest from researchers and industries because of their large overpotential for oxygen evolution in an acidic media, which allows the generation of ^•^OH radicals during a water discharge reaction [20,96]. Studies have shown that the β-PbO_2_ crystal structure is more porous than the α-PbO_2_ crystal structure, and thus the former shows higher catalytic activity and oxidation rate during the AO process [97]. Various elements and oxides such as Fe, Sn, Ti, Sb, Bi, and Co have been added to PbO_2_ as dopants to improve the electrochemical performance of the electrode material [96,98]. These dopants reduce the crystal grain size of the electrode, which results in a higher electroactive surface area and, in turn, higher electrochemical activity [99]. The major limitation of this electrode is the slow leaching of Pb ions into solution, which is a serious concern for wastewater treatment applications and makes the electrode less appealing for industrial scale processes [20,96].

#### 3.2.5. Doped Tin Oxide Electrode

The conductivity of pure SnO_2_ is low but can be tremendously enhanced by doping. Doped SnO_2_ has high conductivity that allows it to function as a suitable and efficient anode in the AO process [20,96]. Antimony (Sb) is the most common dopant for SnO_2_, but it is a toxic substance with an EPA drinking water limit of 6 μg L^−1^ [96]. Alternative dopants such as Ar, B, Bi, F, Cl, and P have been studied as less toxic element for doping SnO_2_ [20,96]. Doped SnO_2_ is a “non-active” electrode that is capable of generating ^•^OH radicals when sufficient potential is applied and exhibits relatively good oxidation potential for the degradation and mineralization of organic pollutants in the AO process. This class of electrodes is not currently common in commercial applications and is less applied for the AO process due to a short service life [100,101]. The short-service life of this electrode is as a result of formation of a non-conducting Sn hydroxide layer at the surface of the anode and passivation of the underlying Ti substrate which causes the delamination of the doped SnO_2_ film. The formation of the Sn hydroxide layer can be mitigated by Pt doping, whereas intercalation of the IrO_2_ layer between the Ti substrate and the doped SnO_2_ film coating has been reported to eliminate the passivation and significantly improve the service life of this electrode [96].

#### 3.2.6. Boron-Doped Diamond Electrode

Thin-film synthetic boron-doped diamond (BDD) electrode is the most efficient and widely studied electrocatalyst material for electrochemical wastewater and soil treatment processes. This electrode is prepared by the chemical vapor deposition method, which is an inexpensive and well-established technology in industry, and thus it is readily available commercially [20,96,102,103]. The wide application of BDD electrodes in many other electrochemistry aspects has assisted the widespread interest and availability of this electrode. Many companies, especially in Europe, specialize in the production of different composition, crystal structure, shape, and size BDD electrodes. The conducting thin-film diamond can be grown on different suitable substrates such as silicon, tungsten, niobium, molybdenum, tantalum, and glassy carbon, but polycrystalline silicon is the traditional substrate for boron-doped diamond thin film because it is able to form a compact self-limiting oxide and has a relatively low electrochemical activity, which prevents film delamination [20,96]. The most common dopant for diamond electrode is boron. The boron doping level in diamond layer expressed as the B/C ratio is about 1000–10,000 ppm [20]. High quality BDD electrodes possess several distinguish technological properties such as extremely high stability under anodic polarization, excellent corrosion resistance in aggressive media, low adsorption property due to inert surface, and wide potential window of application (−1.25–+2.3 V vs. SHE) [20,96]. During electrolysis in the region of water discharge potential, the BDD anode promotes production of weakly adsorbed ^•^OH radicals, which are very reactive and can unselectively and completely mineralize different classes of organic pollutants with a high current efficiency. The BDD anode is also an excellent electrocatalyst for the formation of secondary reactive species such as sulfate radicals, persulfate, and free active chlorine, all of which are relatively strong oxidants that can degrade different classes of organic pollutants [84,86,96].

#### 3.2.7. Doped and Sub-Stoichiometric Titanium Oxides

Conductive Magneli phase sub-stoichiometric TiO_2_ and doped TiO_2_ are recently developed highly promising electrocatalyst materials for electrochemical wastewater treatment. Although stoichiometric TiO_2_ is an insulator with electric conductivity of 10^−9^ Ω^−1^ cm^−1^, its electronic properties can be drastically enhanced either by thermal treatment at above 900 °C under reducing atmosphere (H_2_ or C), which create oxygen deficiencies in its lattice structure or by doping with group five elements such as V, Nb, or Ta [96].

**Figure 6:**
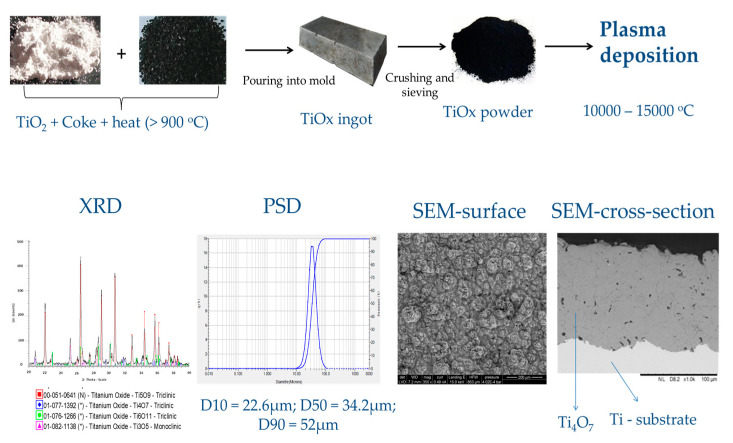
A typical synthesis route for the preparation of plasma elaborated Ti_4_O_7_ and some of its structural properties. Adapted from [18].

Sub-stoichiometric TiO_2_ are a series of conductive oxides known as Magneli phase with the general formula Ti*_n_*O_2*n−*1_ (3 ≤ *n* ≤ 10) [104], Ti_4_O_7_ and T_5_O_9_ being the most conducting and desired of the series. The thermal reduction of TiO_2_ usually yields a mixture of oxides in this series. However, the quality of the electrocatalyst is enhanced by converting the other suboxides of Ti in the mixture to Ti_4_O_9_ and T_5_O_9_. Reduction methods including high temperature (10,000–15,000 °C) plasma elaboration have been tailored to prepare materials that consist primarily of Ti_4_O_7_, with conductivity as high as 166 Ω^−1^ cm^−1^ [18,87,96,104]. Ceramic conductive Magneli phase electrodes which consist primarily of Ti_4_O_7_ are commercially available and, currently, are sold under the trade name Ebonex^®^. However, many laboratories prefer to synthesize this electrode to achieve more controlled properties and composition and enhance the electrocatalytic activity [105]. Both Ebonex^®^ and laboratory synthesized sub-stoichiometric TiO_2_ have been used in different configurations (plate, disc, tubes, flat, and tubular membranes) for electrochemical wastewater treatment [96,106], and studies that have compared Ebonex^®^ with a BDD anode have shown that fewer ^•^OH radicals are generated by the Ebonex^®^ anode but they are more reactive than those formed by a BDD electrode [107]. A typical synthesis route and characteristics of plasma elaborated substoichiometric TiO_2_ is shown in Figure 6.

Niobium doped TiO_2_ (rutile phase) is also a ceramic electrode with very high electric conductivity [96]. The niobium doped rutile (NDR) oxides with the general formula Ti_1−*x*_Nb*_x_*O_2_ (0 ≤ *x* ≤ 0.8) have been studied and found to be conductive [108,109]. Due to the similarity in crystal radii of Nb^5+^ and Ti^4+^, the doping occurs via direct substitution of Nb^5+^ for Ti^4+^, which minimizes the anion deficiencies in the oxides and makes them more resistant to oxidation than Ti_4_O_7_ [110,111]. Both T_4_O_7_ and NDR electrodes have shown promising potential for electrochemical water treatment and both electrode materials can produce ^•^OH radicals via water oxidation [96]. In addition, recent studies have utilized porous T_4_O_7_ electrode as electrochemical membranes or filters (tubular and flat plate membranes) capable of simultaneous filtration and oxidation of organic pollutants [106,112]. However, more studies are still needed on the stability of this electrode and its environmental compatibility. 

## 4. Coupling of SW/EKSR with AO

The effluents of the SW/SF and EKSR processes are loaded with contaminants extracted from the soil. As such, post-treatment decontamination and detoxification of the effluents are needed prior to disposal into the environment. In this frame, selective removal of the contaminants from the effluents could be achieved by combining processes in order to ensure the reusability and recyclability of the extracting solution. Therefore, some studies have employed different remediation processes such as selective electrochemical adsorption [113], photocatalysis [2,3,114,115], Fenton’s reaction based oxidation [2,3,116,117], and electrochemical AOPs [2,12,54] for treatment of effluents of SW/SF and EKSR processes and the possible recycling step of the extracting agents. Among the studied treatment techniques, AO using “non-active” anodes, especially boron-diamond electrode, is an efficient and effective method for both remediation of the contaminant loaded SW/SF and EKSR effluents, as well as recycling of the extracting agents for possible reuse. The following two configurations for coupling AO and a soil remediation process can be proposed: (i) AO as a post-treatment stage, ex situ AO treatment of SW/SF and EKSR effluents and (ii) in situ or simultaneous SW/SF/EKSR with AO treatment. As stated earlier, the former is the most widely investigated configuration and it has been reported by many researchers, whereas the latter is less common in the literature and few works have been performed on it. Details of both configurations are discussed in the following sections.

### 4.1. Ex Situ: Treatment of SW/SF/EKSR Effluent by AO

**Figure 7:**
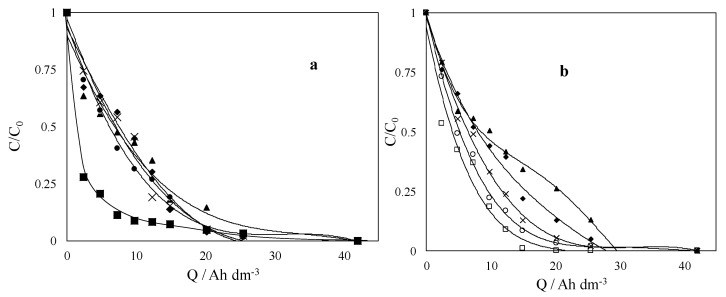
Effect of [SDS]/soil ratio. (▲) 0.5; (♦) 2.5; (●) 5; (×) 12.5; (■) 25, on the degradation profiles of (**a**) SDS and (**b**) atrazine during the CDEO treatment of the SW effluent at a current density of 30 mA cm^−2^. Reprinted with the permission of [122].

The treatment of SW/SF/EKSR effluents by AO is a well investigated combined process for complete removal of contaminants and detoxification of the effluents. AO, using different electrode materials, as well as cell configurations, has been demonstrated to achieve excellent degradation and mineralization of the organic and organo-metallic pollutants contained in SW/SF/EKSR effluents, especially when “non-active” electrodes such as BDD, doped PbO_2_, and SnO_2_ are utilized. In most cases, both the contaminants and the extracting agents contained in the effluents are degraded and mineralized, since ^•^OH radicals are non-selective oxidizing agents, which react with any class of organic pollutants. However, a few studies [2,15] have reported selective degradation of the pollutants encapsulated in the micelles formed by the extracting agents and possible reuse of the extracting agents. Selective degradation was achieved by careful selection of the concentration of the extracting agent (TW80), current density, and supporting electrolyte during the AO process. A lower TW80 concentration reduced it scavenging effect on the generated ^•^OH radicals and allowed faster degradation of the pollutants trapped by the micelles, whereas a high TW80 concentration led to formation of bigger micelles with a diameter larger than the thickness of the BDD(^•^OH) layer at the surface of the electrode, and thus resulted in steric hindrance and lower degradation of pollutants. Additionally, the authors observed faster degradation of targeted pollutants at a low current density as compared with that of extracting agent. The AO treatment in sulfate medium also resulted in faster degradation of TW80 at a higher current density as compared with chlorate medium, which was attributed to the formation of highly oxidant species such as persulfate and sulfate radical, which promoted the oxidation of TW80 in the bulk [15]. Most of the research works on application of AO for treating SW/SF/EKSR effluents have been reported by the Rodrigo group [118,119,120,121]. For instance, dos Santos et al. [120,122] studied the removal of atrazine from soils using combined SW and conductive diamond electrode AO. Atrazine was removed from spiked soils by surfactant fluids-assisted SW and the resulting effluents were treated by using BDD electrolysis. The authors [119] showed that the combined technologies were efficient for removing and totally mineralizing atrazine from soils and SW effluents. The surfactant/soil ratio (Figure 7) was identified as the key parameter for removing atrazine from the soil and it significantly affected the characteristics of the effluents such as the total organic loading and the size of the micelles. In addition to the applied current density, the size of the particles in the SW effluents (reaction media) was the key parameter that influenced the efficiency of the AO process, which continuously decreased during the electrolysis.

The same authors reported the elimination of herbicide oxyfluorfen from SW fluids using either electrolysis [118], sono-electrolysis [119,123], or UV-assisted electrolysis [124] with a BDD electrode. Although the electrolysis using a BDD electrode was quite efficient for the total degradation and mineralization of the herbicide, sono and UV-assisted electrolysis with a BDD electrode achieved faster and better decontamination of the treated effluent. It was demonstrated that prolong sonolysis and UV photolysis treatment without electrolysis could also achieve degradation (with very poor mineralization) of oxyfluorfen but at very slow rate. The same group [125] investigated the treatment of soils polluted with lindane by surfactants aided SW and AO using a BDD electrode. The processes were efficient for removing this hazardous substance from the soil and mineralization from the effluents with over 70% recovery of the surfactant solution after electrolysis for reuse in SW. Effluents of SW containing other contaminants, such as pendimenthalin [126], clopyralid [121], PAHs, and petroleum [127] have also been treated by AO, sono, or irradiation-assisted AO with a BDD electrode. In all cases, electrolysis with a BDD electrode, coupled or not with sonolysis/photolysis, achieved excellent degradation and mineralization of both the herbicides, as well as the surfactants in the washing effluents, and the total decontamination of the effluents could be achieved in 8 h of electrolysis.

**Figure 8:**
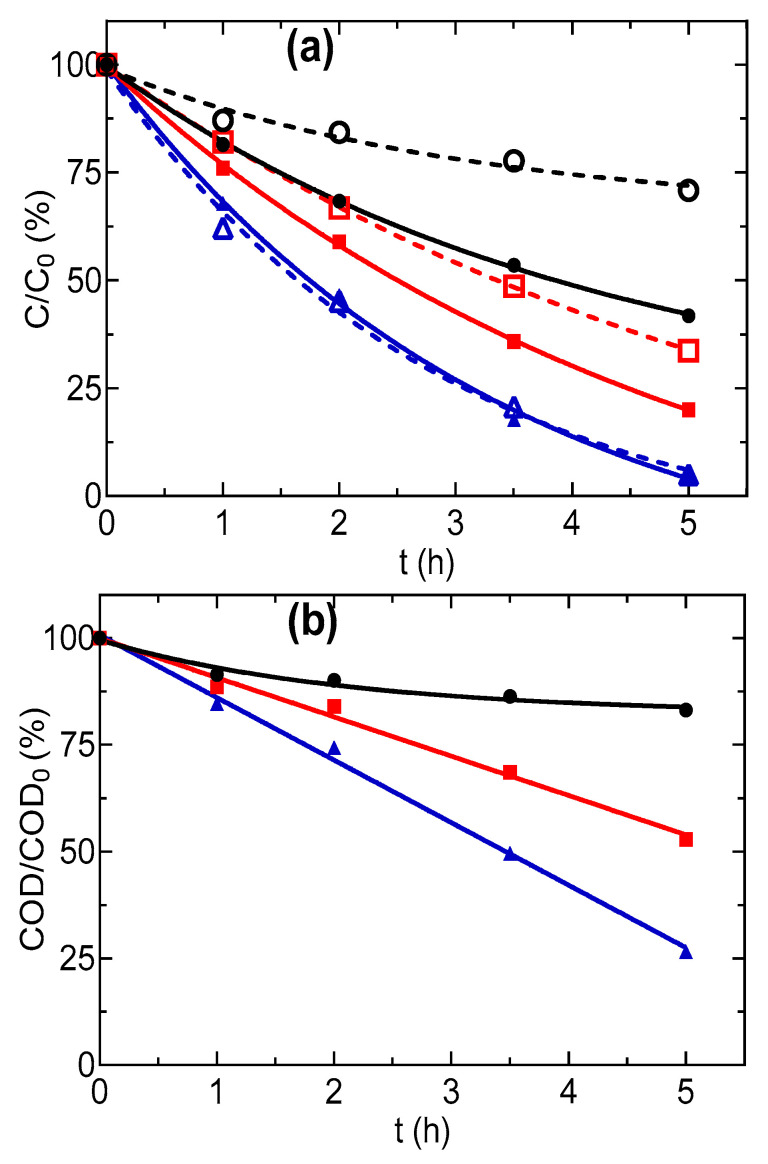
Decay of normalized (**a**) concentration, (○,□,∆) TW80 and (●,■,▲) phenanthrene, and (**b**) COD during the AO of 330 mL SW solution at applied current of (○,●) 200 mA, (□,■) 500 mA, and (∆,▲) 1000 mA. Reprinted with the permission of [16].

Other relevant studies on the treatment of SW/SF effluents by AO have been reported by the Oturan group. For example, a study by [128] investigated the treatment of synthetic SW effluent containing phenanthrene and CD by AO with a BDD electrode. Complete toxicity removal and 100% biodegradability enhancement were achieved during the electrolysis at 1 A, for 8 h. The same group [16] utilized a combination of AO and biological treatment for the elimination of phenanthrene and Tween 80 from synthetic SW solution. AO with a BDD electrode was able to achieve 95% degradation of phenanthrene and Tween 80, as well as 71% COD removal at 1 A, for 5 h (Figure 8). The biological treatment achieved complete phenanthrene and Tween 80 degradation but could only remove 44% COD. Due to the higher energy consumption during AO in this condition, the synergistic effect was achieved by performing AO at a low current and shorter treatment time (3 h) as either pre- or post-treatment to biological process. In this way, a cost-effective combined process was proposed in which AO degraded the organic in the washing solution to biodegradable organic compounds (short-chain carboxylic acids) followed by biological treatment or initial biological treatment of washing solution to remove the biodegradables, followed by AO to degrade recalcitrant organic pollutants remaining in the solution.

**Figure 9:**
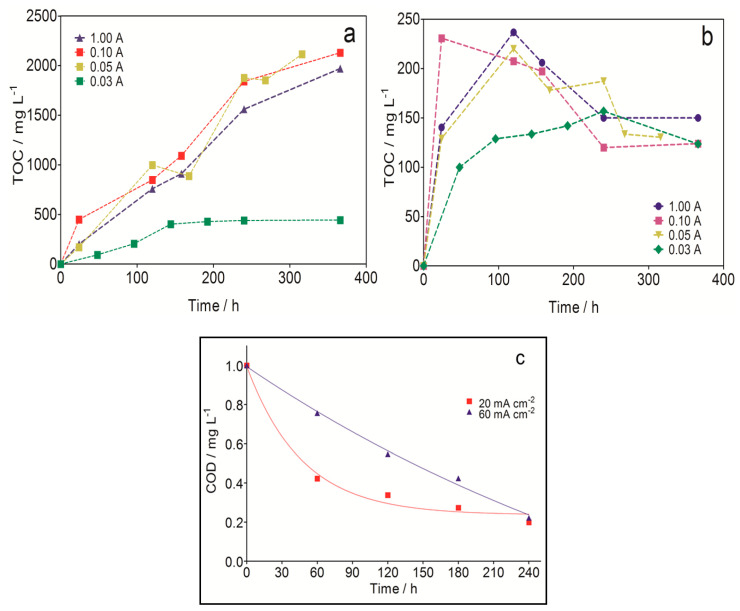
TOC evolution as a function of time at (**a**) anodic and (**b**) cathodic compartments during the electrokinetic soil remediation (EKSR) applying different current values at 25 °C with 0.1 M Na_2_SO_4_ as supporting electrolyte by using graphite electrodes; (**c**) Decay of normalized COD with time obtained during the electrochemical treatment of effluents produced after EKSR of soil polluted with petroleum at applied current density of (■) 20 mA cm^−2^ and (▲) 60 mA cm^−2^. Reprinted with the permission of [129].

Treatment of effluents of EKSR by AO has received less attention than SW/SF effluents possibly because EKSR has been widely utilized for the remediation of heavy metal/toxic anion contaminated soils as compared with organic pollutant contaminated soils. However, a recent study by da Silva et al. (2017) [129] showed the possibility of applying AO using a BDD electrode for the treatment of water produced from EKSR treatment of synthetic petroleum contaminated soil. The EKSR, using graphite electrodes, was able to achieve excellent removal of the petroleum products from the soil as revealed by the gradual accumulation of total organic carbon (TOC) (2250 and 250 mg L^−1^) (Figure 9a,b) in both solutions at the anodic and cathodic chambers, respectively. Electrolytic treatment of the organic loaded effluent of the EKSR using a BDD electrode achieved over 80% COD removal at either 20 or 60 mA cm^−2^ in 4 h, demonstrating the efficacy of the process for the treatment of the effluent.

### 4.2. In Situ: Treatment of SW Effluent by AO

**Figure 10:**
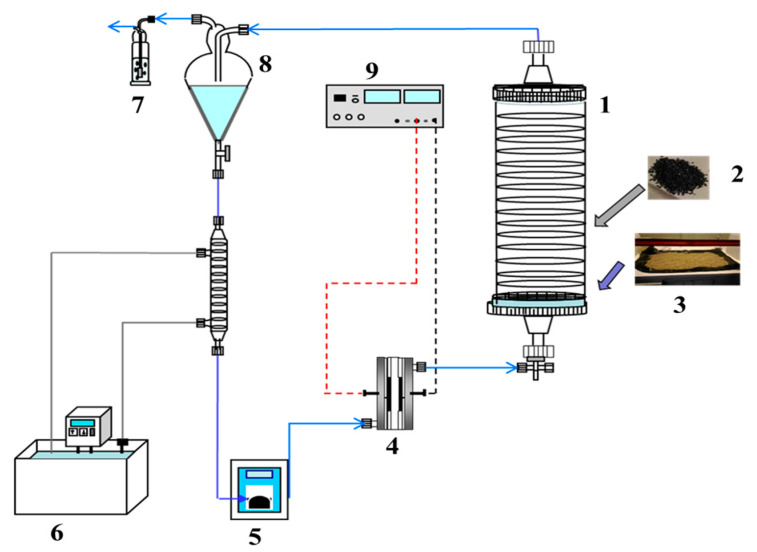
Schematic of concurrent SW/zero valent iron (ZVI) dehalogenation and electrochemical oxidation processes for remediation of soil contaminated by herbicide clopyralid. (**1**) SW reactor; (**2**) ZVI; (**3**) Contaminated soil; (**4**) Electrochemical reactor; (**5**) Peristatic pump; (**6**) Thermostatic cooling system; (**7**) Secondary tank; (**8**) Gas exchange; (**9**) DC power generator. Adapted from [130].

This configuration involves performing SW/SF/EKSR and AO simultaneously for remediation of organic pollutant contaminated soils. This approach is very rare in the literature, but the concept is very exciting for field applications. The electrolyte chambers of the EKSR or flushing fluid channels serve as the electrolytic cell where the electrolysis process is carried out. Some challenges of this configuration include agitation problem, possibility of wearing of the electrode due to friction impacted by the soil particles, sampling and longer treatment time especially in SW due to the time required for an efficient washing process. As stated earlier, in situ AO with SW/SF/EKSR has not been extensively explored, but a recent study by the Rodrigo group [130] reported the combination of SW, zero valent iron (ZVI) dehalogenation, and AO in a single assemble reactor for removing and degrading clopyralid in spiked contaminated soil. The assemble consisted of the electrochemical cell, the SW, and the dehalogenation tank made of a rigid silicon tube of 1 m × 69 mm, and continuously circulated with 2 L of washing solution at 40 dm^3^ h^−1^ (Figure 10). The electrochemical cell was equipped with a BDD anode and a stainless steel cathode, both of geometric surface area of 78 cm^2^. The combined treatment was efficient, achieving complete removal of the chlorinated organics from the soil and complete mineralization of the organics in the generated liquid waste effluent during AO at 25 mA cm^−2^. The authors observed that the combined process of SW and AO achieved similar efficiency as compared with a system operated with iron addition, and therefore ZVI dehalogenation may not be necessary for the remediation of the clopyralid.

## 5. Future Perspective and Concluding Remarks

Combined SW/SF/EKSR and AO using a BDD electrode is an exciting technology that has been studied for remediation of different organic pollutant contaminated soils, with very promising results obtained for bench and some pilot scale experiments. However, extensive studies are still required to advance this technology for possible license and commercialization. Future studies should be tailored towards the optimization of operational parameters, modeling and automation, toxicity assessment, environmental and economic analysis, field testing, as well as the potential of reuse of both the remediated soil and effluents for other purposes (i.e., agriculture and construction). The operation parameters that influence both the soil SW/SF/EKSR (such as physicochemical properties of the soils, nature of the contaminants and contamination levels, washing solution composition and concentration, as well as washing solution/soil ratio) and AO (such as current density, electrode materials, and inorganic ions in the treated solution) require proper optimization to ensure optimum efficiency at the lowest economic cost possible. Currently, both technologies have been separately studied which have allowed us to understand the main parameters that influence the decontamination efficacy of each one of them. For AO, the existing literature has demonstrated that each effluent contaminated with specific pollutant or a mixture of pollutants is a particular case, and its treatment depends on several operating conditions (which must be strongly considered during scale-up of the process); nevertheless, the use of non-active anodes guarantees higher decontamination levels by producing higher concentrations of oxidizing agents. Meanwhile, SW/SF/EKSR processes depend mainly on the chemical properties of the pollutants, as well as the physical-chemical soil conditions. For this reason, the effects of the supporting electrolytes and their composition play a key role in the in situ or ex situ decontamination steps for removing organic/inorganic pollutants from the soil because combined with the chemical/electrochemical phenomena, several changes of the pH, dissolved cationic or anionic species, temperature, viscosity, flow directions, and so on, can be attained. During optimization and scale-up of the combine SW/SF/EKSR and AO technologies, problems or challenges associated with each parameter, such as high organic loading and cost associated with the use of a higher washing solution/soil ratio during the SW/SF process, heating and hydrogen evolution at a higher current density, as well as non-uniform current distribution in a large surface area electrode during AO, should also be carefully considered.

Toxicity assessment and environmental impact of the combine technologies on the ecosystem should be thoroughly studied, since the literature currently available on the combine processes paid little attention to this aspect. Since both the treated soil and washing effluents are disposed into the environments, thorough assessment of the toxicity of both soils and effluents are necessary to avoid secondary pollution and hazardous effect on the ecosystem. Additionally, the economic assessments of the technologies are necessary in order to compare the benefit and disadvantage of the combine technologies with other existing soil remediation processes. Extensive studies on scale-up/pilot and field experiments are still needed before the full-scale implementation of the combine technologies. Most of the studies reported in literature are either bench or pre-pilot scales, as such pilot and field studies are essential before the certification of the process because future developments rely upon the close collaboration of analytical chemists, engineers, and geologists to ensure effective application and exploitation of new electrochemical environmental applications [131].

Conclusively, SW/SF/EK/SR coupled with AO is an efficient technology for the remediation of organic contaminated soils. The AO process can be applied as a post-treatment stage to treat the organic loads in the effluents obtained after SW/SF/EKSR processes. In situ SW/SF/EK/SR coupled with AO is very rare in the literature but the configuration is a very exciting technology for treating organic polluted soils. Future studies should be channeled towards optimization of the operation parameters, assessment of toxicity and environmental impact of the treated soil and washing effluents, as well as scale-up and field implementation of the combine process. Several other complementary techniques or materials are emerging that can provide improvements in the efficacy of integrated technologies (e.g., nanomaterials [132] and absorbents [133]).

## References

[1] Ganiyu S.O., van Hullebusch E.D., Cretin M., Esposito G., Oturan M.A. (2015). Coupling of membrane filtration and advanced oxidation processes for removal of pharmaceutical residues: A critical review. Sep. Purif. Technol..

[2] Trellu C., Mousset E., Pechaud Y., Huguenot D., van Hullebusch E.D., Esposito G., Oturan M.A. (2016). Removal of hydrophobic organic pollutants from soil washing/flushing solutions: A critical review. J. Hazard. Mater..

[3] Cheng M., Zeng G., Huang D., Lai C., Xu P., Zhang C., Liu Y. (2016). Hydroxyl radicals based advanced oxidation processes (AOPs) for remediation of soils contaminated with organic compounds: A review. Chem. Eng. J..

[4] Manz M., Wenzel K.D., Dietze U., Schüürmann G. (2001). Persistent organic pollutants in agricultural soils of central Germany. Sci. Total Environ..

[5] Tabak H.H., Lens P., van Hullebusch E.D., Dejonghe W. (2005). Developments in Bioremediation of Soils and Sediments Polluted with Metals and Radionuclides—1. Microbial Processes and Mechanisms Affecting Bioremediation of Metal Contamination and Influencing Metal Toxicity and Transport. Rev. Environ. Sci. Biotechnol..

[6] Mao X., Jiang R., Xiao W., Yu J. (2015). Use of surfactants for the remediation of contaminated soils: A review. J. Hazard. Mater..

[7] Wuana R.A., Okieimen F.E. (2011). Heavy Metals in Contaminated Soils: A Review of Sources, Chemistry, Risks and Best Available Strategies for Remediation. Int. Sch. Res. Not..

[8] Arias-Estévez M., López-Periago E., Martínez-Carballo E., Simal-Gándara J., Mejuto J.C., García-Río L. (2008). The mobility and degradation of pesticides in soils and the pollution of groundwater resources. Agric. Ecosyst. Environ..

[9] Villa R.D., Trovó A.G., Nogueira R.F.P. (2008). Environmental implications of soil remediation using the Fenton process. Chemosphere.

[10] He X., de la Cruz A.A., O’Shea K.E., Dionysiou D.D. (2014). Kinetics and mechanisms of cylindrospermopsin destruction by sulfate radical-based advanced oxidation processes. Water Res..

[11] Roudier P. (2005). Techniques de réhabilitation des sites et sols pollués—Fiches de synthèse. Trav. Publics Infrastruct..

[12] Rodrigo M.A., Oturan N., Oturan M.A. (2014). Electrochemically Assisted Remediation of Pesticides in Soils and Water: A Review. Chem. Rev..

[13] Lau E.V., Gan S., Ng H.K., Poh P.E. (2014). Extraction agents for the removal of polycyclic aromatic hydrocarbons (PAHs) from soil in soil washing technologies. Environ. Pollut..

[14] Mousset E., Oturan E.D.V., Hullebusch G., Guibaud G. (2014). Esposito, Soil Washing/Flushing Treatments of Organic Pollutants Enhanced by Cyclodextrins and Integrated Treatments: State of the Art. Crit. Rev. Environ. Sci. Technol..

[15] Trellu C., Oturan N., Pechaud Y., van Hullebusch E.D., Esposito G., Oturan M.A. (2017). Anodic oxidation of surfactants and organic compounds entrapped in micelles—Selective degradation mechanisms and soil washing solution reuse. Water Res..

[16] Trellu C., Ganzenko O., Papirio S., Pechaud Y., Oturan N., Huguenot D., van Hullebusch E.D., Esposito G., Oturan M.A. (2016). Combination of anodic oxidation and biological treatment for the removal of phenanthrene and Tween 80 from soil washing solution. Chem. Eng. J..

[17] Huang D., Hu C., Zeng G., Cheng M., Xu P., Gong X., Wang R., Xue W. (2017). Combination of Fenton processes and biotreatment for wastewater treatment and soil remediation. Sci. Total Environ..

[18] Ganiyu S.O., Oturan N., Raffy S., Cretin M., Esmilaire R., van Hullebusch E., Esposito G., Oturan M.A. (2016). Sub-stoichiometric titanium oxide (Ti_4_O_7_) as a suitable ceramic anode for electrooxidation of organic pollutants: A case study of kinetics, mineralization and toxicity assessment of amoxicillin. Water Res..

[19] Brito C.N., Ferreira M.B., Marcionilio S.M.L.D.O., Santos E.C.M.D.M., León J.J.L., Ganiyu S.O., Martínez-Huitle C.A. (2018). Electrochemical Oxidation of Acid Violet 7 Dye by Using Si/BDD and Nb/BDD Electrodes. J. Electrochem. Soc..

[20] Panizza M., Cerisola G. (2009). Direct and mediated anodic oxidation of organic pollutants. Chem. Rev..

[21] Martínez-Huitle C.A., Rodrigo M.A., Sirés I., Scialdone O. (2015). Single and Coupled Electrochemical Processes and Reactors for the Abatement of Organic Water Pollutants: A Critical Review. Chem. Rev..

[22] Martínez-Huitle C.A., Ferro S. (2006). Electrochemical oxidation of organic pollutants for the wastewater treatment: Direct and indirect processes. Chem. Soc. Rev..

[23] Ganiyu S.O., Huong Le T.X., Bechelany M., Oturan N., Papirio S., Esposito G., van Hullebusch E., Cretin M., Oturan M.A. (2018). Electrochemical mineralization of sulfamethoxazole over wide pH range using FeIIFeIII LDH modified carbon felt cathode: Degradation pathway, toxicity and reusability of the modified cathode. Chem. Eng. J..

[24] Ganiyu S.O., Gamal El-Din M. (2020). Insight into in-situ radical and non-radical oxidative degradation of organic compounds in complex real matrix during electrooxidation with boron doped diamond electrode: A case study of oil sands process water treatment. Appl. Catal. B Environ..

[25] Sirés I., Brillas E., Oturan M.A., Rodrigo M.A., Panizza M. (2014). Electrochemical advanced oxidation processes: Today and tomorrow. A review. Environ. Sci. Pollut. Res..

[26] Martínez-Huitle C.A., Panizza M. (2018). Electrochemical oxidation of organic pollutants for wastewater treatment. Curr. Opin. Electrochem..

[27] Durán F.E., de Araújo D.M., do Nascimento Brito C., Santos E.V., Ganiyu S.O., Martínez-Huitle C.A. (2018). Electrochemical technology for the treatment of real washing machine effluent at pre-pilot plant scale by using active and non-active anodes. J. Electroanal. Chem..

[28] Ganiyu S.O., Oturan N., Raffy S., Esposito G., van Hullebusch E.D., Cretin M., Oturan M.A. (2017). Use of Sub-stoichiometric Titanium Oxide as a Ceramic Electrode in Anodic Oxidation and Electro-Fenton Degradation of the Beta-blocker Propranolol: Degradation Kinetics and Mineralization Pathway. Electrochim. Acta.

[29] Sirés I., Brillas E. (2012). Remediation of water pollution caused by pharmaceutical residues based on electrochemical separation and degradation technologies: A review. Environ. Int..

[30] Särkkä H., Bhatnagar A., Sillanpää M. (2015). Recent developments of electro-oxidation in water treatment—A review. J. Electroanal. Chem..

[31] Radjenovic J., Sedlak D. (2015). Challenges and opportunities for electrochemical processes as next generation technologies for the treatment of contaminated water. Environ. Sci. Technol..

[32] Moreira F.C., Boaventura R.A.R., Brillas E., Vilar V.J.P. (2017). Electrochemical advanced oxidation processes: A review on their application to synthetic and real wastewaters. Appl. Catal. B Environ..

[33] Oturan M.A. (2014). Electrochemical advanced oxidation technologies for removal of organic pollutants from water. Environ. Sci. Pollut. Res..

[34] Oturan M.A., Aaron J.J. (2014). Advanced Oxidation Processes in Water/Wastewater Treatment: Principles and Applications. A Review. Crit. Rev. Environ. Sci. Technol..

[35] Vieira dos Santos E., Souza F., Saez C., Cañizares P., Lanza M.R.V., Martinez-Huitle C.A., Rodrigo M.A. (2016). Application of electrokinetic soil flushing to four herbicides: A comparison. Chemosphere.

[36] Mulligan C.N., Yong R.N., Gibbs B.F. (2001). Surfactant-enhanced remediation of contaminated soil: A review. Eng. Geol..

[37] Atteia O., Del Campo Estrada E., Bertin H. (2013). Soil flushing: A review of the origin of efficiency variability. Rev. Environ. Sci. Biotechnol..

[38] Yang K., Zhu L., Xing B. (2006). Enhanced Soil Washing of Phenanthrene by Mixed Solutions of TX100 and SDBS. Environ. Sci. Technol..

[39] Park S.J., Back J.M. (2000). Prediction of partition coefficients for some organic compounds using UNIFAC. J. Ind. Eng. Chem..

[40] Khodadoust A.P., Bagchi R., Suidan M.T., Brenner R.C., Sellers N.G. (2000). Removal of PAHs from highly contaminated soils found at prior manufactured gas operations. J. Hazard. Mater..

[41] Lee P.H., Ong S.K., Golchin J., Nelson G.L. (2001). Use of solvents to enhance PAH biodegradation of coal tar. Water Res..

[42] Vishnyakov A., Lee M.T., Neimark A.V. (2013). Prediction of the Critical Micelle Concentration of Nonionic Surfactants by Dissipative Particle Dynamics Simulations. J. Phys. Chem. Lett..

[43] Lee M.T., Vishnyakov A., Neimark A.V. (2013). Calculations of Critical Micelle Concentration by Dissipative Particle Dynamics Simulations: The Role of Chain Rigidity. J. Phys. Chem. B.

[44] Kim B.K., Baek K., Ko S.H., Yang J.W. (2011). Research and field experiences on electrokinetic remediation in South Korea. Sep. Purif. Technol..

[45] Nguyen T.T., Youssef N.H., McInerney M.J., Sabatini D.A. (2008). Rhamnolipid biosurfactant mixtures for environmental remediation. Water Res..

[46] Lai C.C., Huang Y.C., Wei Y.H., Chang J.S. (2009). Biosurfactant-enhanced removal of total petroleum hydrocarbons from contaminated soil. J. Hazard. Mater..

[47] Zhao B., Zhu L., Gao Y. (2005). A novel solubilization of phenanthrene using Winsor I microemulsion-based sodium castor oil sulfate. J. Hazard. Mater..

[48] Bragato M., El Seoud O.A. (2003). Formation, properties, and “ex situ” soil decontamination by vegetable oil-based microemulsions. J. Surfactants Deterg..

[49] Song G., Lu C., Lin J.M. (2007). Application of surfactants and microemulsions to the extraction of pyrene and phenanthrene from soil with three different extraction methods. Anal. Chim. Acta.

[50] Badr T., Hanna K., de Brauer C. (2004). Enhanced solubilization and removal of naphthalene and phenanthrene by cyclodextrins from two contaminated soils. J. Hazard. Mater..

[51] Viglianti C., Hanna K., de Brauer C., Germain P. (2006). Removal of polycyclic aromatic hydrocarbons from aged-contaminated soil using cyclodextrins: Experimental study. Environ. Pollut..

[52] Petitgirard A., Djehiche M., Persello J., Fievet P., Fatin-Rouge N. (2009). PAH contaminated soil remediation by reusing an aqueous solution of cyclodextrins. Chemosphere.

[53] Szejtli J. (1989). Downstream processing using cyclodextrins. Trends Biotechnol..

[54] Gómez J., Alcántara M.T., Pazos M., Sanromán M.Á. (2010). Soil washing using cyclodextrins and their recovery by application of electrochemical technology. Chem. Eng. J..

[55] Li Z., Chen J., Yang J., Su Y., Fan X., Wu Y., Yu C., Wang Z.L. (2015). β-cyclodextrin enhanced triboelectrification for self-powered phenol detection and electrochemical degradation. Energy Environ. Sci..

[56] Yang C., Zeng Q., Wang Y., Liao B., Sun J., Shi H., Chen X. (2010). Simultaneous elution of polycyclic aromatic hydrocarbons and heavy metals from contaminated soil by two amino acids derived from β-cyclodextrins. J. Environ. Sci..

[57] Sánchez-Trujillo M.A., Morillo E., Villaverde J., Lacorte S. (2013). Comparative effects of several cyclodextrins on the extraction of PAHs from an aged contaminated soil. Environ. Pollut..

[58] Conte P., Zena A., Pilidis G., Piccolo A. (2001). Increased retention of polycyclic aromatic hydrocarbons in soils induced by soil treatment with humic substances. Environ. Pollut..

[59] Conte P., Agretto A., Spaccini R., Piccolo A. (2005). Soil remediation: Humic acids as natural surfactants in the washings of highly contaminated soils. Environ. Pollut..

[60] Li W., Zhu X., He Y., Xing B., Xu J., Brookes P.C. (2013). Enhancement of water solubility and mobility of phenanthrene by natural soil nanoparticles. Environ. Pollut..

[61] Ling W., Ren L., Gao Y., Zhu X., Sun B. (2009). Impact of low-molecular-weight organic acids on the availability of phenanthrene and pyrene in soil. Soil Biol. Biochem..

[62] Parrish C.C. (1988). Dissolved and particulate marine lipid classes: A review. Mar. Chem..

[63] Gong Z., Wilke B.M., Alef K., Li P. (2005). Influence of soil moisture on sunflower oil extraction of polycyclic aromatic hydrocarbons from a manufactured gas plant soil. Sci. Total Environ..

[64] Gong Z., Wilke B.M., Alef K., Li P., Zhou Q. (2006). Removal of polycyclic aromatic hydrocarbons from manufactured gas plant-contaminated soils using sunflower oil: Laboratory column experiments. Chemosphere.

[65] Gong Z., Alef K., Wilke B.M., Li P. (2007). Activated carbon adsorption of PAHs from vegetable oil used in soil remediation. J. Hazard. Mater..

[66] Lau E.V., Gan S., Ng H.K. (2012). Extraction of phenanthrene and fluoranthene from contaminated sand using palm kernel and soybean oils. J. Environ. Manag..

[67] Yeung A.T. (2005). Contaminant Extractability by Electrokinetics. Environ. Eng. Sci..

[68] Yeung A.T., Gu Y.Y. (2011). A review on techniques to enhance electrochemical remediation of contaminated soils. J. Hazard. Mater..

[69] Mary M., Christopher L. (2002). Electroremediation of Contaminated Soils. J. Environ. Eng..

[70] Gomes H.I., Dias-Ferreira C., Ribeiro A.B. (2012). Electrokinetic remediation of organochlorines in soil: Enhancement techniques and integration with other remediation technologies. Chemosphere.

[71] Reddy Krishna R., Saichek Richard E. (2003). Effect of Soil Type on Electrokinetic Removal of Phenanthrene Using Surfactants and Cosolvents. J. Environ. Eng..

[72] Reddy K.R., Chinthamreddy S. (2004). Enhanced Electrokinetic Remediation of Heavy Metals in Glacial Till Soils Using Different Electrolyte Solutions. J. Environ. Eng..

[73] Reddy K.R., Maturi K., Cameselle C. (2009). Sequential Electrokinetic Remediation of Mixed Contaminants in Low Permeability Soils. J. Environ. Eng..

[74] López-Vizcaíno R., Sáez C., Mena E., Villaseñor J., Cañizares P., Rodrigo M.A. (2011). Electro-osmotic fluxes in multi-well electro-remediation processes. J. Environ. Sci. Health Part A.

[75] Lopez-Vizcaíno R., Sáez C., Cañizares P., Rodrigo M.A. (2012). Electrocoagulation of the effluents from surfactant-aided soil-remediation processes. Sep. Purif. Technol..

[76] López-Vizcaíno R., Sáez C., Cañizares P., Rodrigo M.A. (2012). The use of a combined process of surfactant-aided soil washing and coagulation for PAH-contaminated soils treatment. Sep. Purif. Technol..

[77] Yuan S., Tian M., Lu X. (2006). Electrokinetic movement of hexachlorobenzene in clayed soils enhanced by Tween 80 and β-cyclodextrin. J. Hazard. Mater..

[78] Yuan S., Wan J., Lu X. (2007). Electrokinetic movement of multiple chlorobenzenes in contaminated soils in the presence of β-cyclodextrin. J. Environ. Sci..

[79] Jackman S.A., Maini G., Sharman A.K., Sunderland G., Knowles C.J. (2001). Electrokinetic movement and biodegradation of 2,4-dichlorophenoxyacetic acid in silt soil. Biotechnol. Bioeng..

[80] Ma J.W., Wang F.Y., Huang Z.H., Wang H. (2010). Simultaneous removal of 2,4-dichlorophenol and Cd from soils by electrokinetic remediation combined with activated bamboo charcoal. J. Hazard. Mater..

[81] Ribeiro A.B., Mateus E.P., Rodríguez-Maroto J.M. (2011). Removal of organic contaminants from soils by an electrokinetic process: The case of molinate and bentazone. Experimental and modeling. Sep. Purif. Technol..

[82] Oonnittan A., Shrestha R.A., Sillanpää M. (2009). Effect of cyclodextrin on the remediation of hexachlorobenzene in soil by electrokinetic Fenton process. Sep. Purif. Technol..

[83] Oonnittan A., Isosaari P., Sillanpää M. (2010). Oxidant availability in soil and its effect on HCB removal during electrokinetic Fenton process. Sep. Purif. Technol..

[84] Ganiyu S.O., Martínez-Huitle C.A. (2019). Nature, Mechanisms and Reactivity of Electrogenerated Reactive Species at Thin-Film Boron-Doped Diamond (BDD) Electrodes during Electrochemical Wastewater Treatment. ChemElectroChem.

[85] Rodrigo M.A., Cañizares P., Buitrón C., Sáez C. (2010). Electrochemical technologies for the regeneration of urban wastewaters. Electrochim. Acta.

[86] Ganiyu S.O., Martínez-Huitle C.A., Rodrigo M.A. (2020). Renewable energies driven electrochemical wastewater/soil decontamination technologies: A critical review of fundamental concepts and applications. Appl. Catal. B Environ..

[87] Ganiyu S.O., Oturan N., Trellu C., Raffy S., Cretin M., Causserand C., Oturan M.A. (2019). Electrochemical Abatement of Analgesic Antipyretic 4-Aminophenazone using Conductive Boron-Doped Diamond and Sub-Stoichiometric Titanium Oxide Anodes: Kinetics, Mineralization and Toxicity Assessment. ChemElectroChem.

[88] Yi F., Chen S., Yuan C. (2008). Effect of activated carbon fiber anode structure and electrolysis conditions on electrochemical degradation of dye wastewater. J. Hazard. Mater..

[89] Abdalrhman A.S., Ganiyu S.O., Gamal El-Din M. (2019). Degradation kinetics and structure-reactivity relation of naphthenic acids during anodic oxidation on graphite electrodes. Chem. Eng. J..

[90] Rueffer M., Bejan D., Bunce N.J. (2011). Graphite: An active or an inactive anode?. Electrochim. Acta.

[91] de Moura D.C., de Araújo C.K.C., Zanta C.L.P.S., Salazar R., Martínez-Huitle C.A. (2014). Active chlorine species electrogenerated on Ti/Ru_0.3_Ti_0.7_O_2_ surface: Electrochemical behavior, concentration determination and their application. J. Electroanal. Chem..

[92] Santos M.J.R., Medeiros M.C., Oliveira T.M.B.F., Morais C.C.O., Mazzetto S.E., Martínez-Huitle C.A., Castro S.S.L. (2016). Electrooxidation of cardanol on mixed metal oxide (RuO_2_-TiO_2_ and IrO_2_-RuO_2_-TiO_2_) coated titanium anodes: Insights into recalcitrant phenolic compounds. Electrochim. Acta.

[93] Ganiyu S.O., Oturan N., Raffy S., Cretin M., Causserand C., Oturan M.A. (2018). Efficiency of plasma elaborated sub-stoichiometric titanium oxide (Ti_4_O_7_) ceramic electrode for advanced electrochemical degradation of paracetamol in different electrolyte media. Sep. Purif. Technol..

[94] Brillas E., Garcia-Segura S., Skoumal M., Arias C. (2010). Electrochemical incineration of diclofenac in neutral aqueous medium by anodic oxidation using Pt and boron-doped diamond anodes. Chemosphere.

[95] Rodgers J.D., Jedral W., Bunce N.I. (1999). Electrochemical oxidation of chlorinated phenols. Environ. Sci. Technol..

[96] Chaplin B.P. (2014). Critical review of electrochemical advanced oxidation processes for water treatment applications. Environ. Sci. Process. Impacts.

[97] Abaci S., Tamer U., Pekmez K., Yildiz A. (2005). Performance of different crystal structures of PbO_2_ on electrochemical degradation of phenol in aqueous solution. Appl. Surf. Sci..

[98] Santos J.E.L., Quiroz M.A., Cerro-Lopez M., de Moura D.C., Martínez-Huitle C.A. (2018). Evidence for the electrochemical production of persulfate at TiO_2_ nanotubes decorated with PbO_2_. New J. Chem..

[99] Liu H., Liu Y., Zhang C., Shen R. (2008). Electrocatalytic oxidation of nitrophenols in aqueous solution using modified PbO_2_ electrodes. J. Appl. Electrochem..

[100] Correa-Lozano B., Comninellis C., De Battisti A. (1997). Service life of Ti/SnO_2_–Sb_2_O_5_ anodes. J. Appl. Electrochem..

[101] Lipp L., Pletcher D. (1997). The preparation and characterization of tin dioxide coated titanium electrodes. Electrochim. Acta.

[102] Martínez-Huitle C.A., dos Santos E.V., de Araújo D.M., Panizza M. (2012). Applicability of diamond electrode/anode to the electrochemical treatment of a real textile effluent. J. Electroanal. Chem..

[103] Trellu C., Chakraborty S., Nidheesh P.V., Oturan M.A. (2019). Environmental Applications of Boron-Doped Diamond Electrodes: 2. Soil Remediation and Sensing Applications. ChemElectroChem.

[104] Walsh F.C., Wills R.G.A. (2010). The continuing development of Magnéli phase titanium sub-oxides and Ebonex^®^ electrodes. Electrochim. Acta.

[105] Hayfield P.C.S. (2001). Development of a New Material.

[106] Trellu C., Coetsier C., Rouch J.-C., Esmilaire R., Rivallin M., Cretin M., Causserand C. (2018). Mineralization of organic pollutants by anodic oxidation using reactive electrochemical membrane synthesized from carbothermal reduction of TiO_2_. Water Res..

[107] Bejan D., Guinea E., Bunce N.J. (2012). On the nature of the hydroxyl radicals produced at boron-doped diamond and Ebonex^®^ anodes. Electrochim. Acta.

[108] Chen G., Waraksa C.C., Cho H., Macdonald D.D., Mallouka T.E. (2003). EIS Studies of Porous Oxygen Electrodes with Discrete Particles. J. Electrochem. Soc..

[109] Chen G., Bare S.R., Mallouk T.E. (2002). Development of Supported Bifunctional Electrocatalysts for Unitized Regenerative Fuel Cells. J. Electrochem. Soc..

[110] Morris D., Dou Y., Rebane J., Mitchell C.E.J., Egdell R.G., Law D.S.L., Vittadini A., Casarin M. (2000). Photoemission and STM study of the electronic structure of Nb-doped TiO_2_. Phys. Rev. B.

[111] Lee H.-Y., Robertson J. (2013). Doping and compensation in Nb-doped anatase and rutile TiO_2_. J. Appl. Phys..

[112] Zaky A.M., Chaplin B.P. (2013). Porous Substoichiometric TiO_2_ Anodes as Reactive Electrochemical Membranes for Water Treatment. Environ. Sci. Technol..

[113] Cotillas S., Sáez C., Cañizares P., Cretescu I., Rodrigo M.A. (2018). Removal of 2,4-D herbicide in soils using a combined process based on washing and adsorption electrochemically assisted. Sep. Purif. Technol..

[114] Zhou Z., Zhang Y., Wang H., Chen T., Lu W. (2014). The comparative photodegradation activities of pentachlorophenol (PCP) and polychlorinated biphenyls (PCBs) using UV alone and TiO_2_-derived photocatalysts in methanol soil washing solution. PLoS ONE.

[115] Zhu X., Zhou D., Wang Y., Cang L., Fang G., Fan J. (2012). Remediation of polychlorinated biphenyl-contaminated soil by soil washing and subsequent TiO_2_ photocatalytic degradation. J. Soils Sediments.

[116] Lindsey M.E., Xu G., Lu J., Tarr M.A. (2003). Enhanced Fenton degradation of hydrophobic organics by simultaneous iron and pollutant complexation with cyclodextrins. Sci. Total Environ..

[117] Manzano M.A., Perales J.A., Sales D., Quiroga J.M. (2004). Catalyzed Hydrogen Peroxide Treatment of Polychlorinated Biphenyl Contaminated Sandy Soils. Water Air Soil Pollut..

[118] dos Santos E.V., Sáez C., Martínez-Huitle C.A., Cañizares P., Rodrigo M.A. (2016). Removal of oxyfluorfen from ex-situ soil washing fluids using electrolysis with diamond anodes. J. Environ. Manag..

[119] dos Santos E.V., Sáez C., Cañizares P., Rodrigo M.A., Martínez-Huitle C.A. (2018). Coupling Photo and Sono Technologies with BDD Anodic Oxidation for Treating Soil-Washing Effluent Polluted with Atrazine. J. Electrochem. Soc..

[120] dos Santos E.V., Sáez C., Martínez-Huitle C.A., Cañizares P., Rodrigo M.A. (2015). The role of particle size on the conductive diamond electrochemical oxidation of soil-washing effluent polluted with atrazine. Electrochem. Commun..

[121] Martín de Vidales M.J., Castro M.P., Sáez C., Cañizares P., Rodrigo M.A. (2019). Radiation-assisted electrochemical processes in semi-pilot scale for the removal of clopyralid from soil washing wastes. Sep. Purif. Technol..

[122] dos Santos E.V., Sáez C., Martínez-Huitle C.A., Cañizares P., Rodrigo M.A. (2015). Combined soil washing and CDEO for the removal of atrazine from soils. J. Hazard. Mater..

[123] Santos E.V.D., Sáez C., Cañizares P., Martínez-Huitle C.A., Rodrigo M.A. (2017). Treating soil-washing fluids polluted with oxyfluorfen by sono-electrolysis with diamond anodes. Ultrason. Sonochem..

[124] Dos Santos E.V., Sáez C., Cañizares P., Martínez-Huitle C.A., Rodrigo M.A. (2017). UV assisted electrochemical technologies for the removal of oxyfluorfen from soil washing wastes. Chem. Eng. J..

[125] Muñoz-Morales M., Braojos M., Sáez C., Cañizares P., Rodrigo M.A. (2017). Remediation of soils polluted with lindane using surfactant-aided soil washing and electrochemical oxidation. J. Hazard. Mater..

[126] Almazán-Sánchez P.T., Cotillas S., Sáez C., Solache-Ríos M.J., Martínez-Miranda V., Cañizares P., Linares-Hernández I., Rodrigo M.A. (2017). Removal of pendimethalin from soil washing effluents using electrolytic and electro-irradiated technologies based on diamond anodes. Appl. Catal. B Environ..

[127] Dos Santos E.V., Sáez C., Cañizares P., da Silva D.R., Martínez-Huitle C.A., Rodrigo M.A. (2017). Treatment of ex-situ soil-washing fluids polluted with petroleum by anodic oxidation, photolysis, sonolysis and combined approaches. Chem. Eng. J..

[128] Mousset E., Oturan N., van Hullebusch E.D., Guibaud G., Esposito G., Oturan M.A. (2014). Treatment of synthetic soil washing solutions containing phenanthrene and cyclodextrin by electro-oxidation. Influence of anode materials on toxicity removal and biodegradability enhancement. Appl. Catal. B Environ..

[129] Bernardo Sabino da Silva E. (2017). Electrokinetic Treatment of Polluted Soil with Petroleum Coupled to an Advanced Oxidation Process for Remediation of Its Effluent. Int. J. Electrochem. Sci..

[130] Carvalho de Almeida C., Muñoz-Morales M., Sáez C., Cañizares P., Martínez-Huitle C.A., Rodrigo M.A. (2019). Integrating ZVI-dehalogenation into an electrolytic soil-washing cell. Sep. Purif. Technol..

[131] Ganiyu S.O., Martínez-Huitle C.A. (2020). The use of renewable energies driving electrochemical technologies for environmental applications. Curr. Opin. Electrochem..

[132] Gładysz-Płaska A., Skwarek E., Budnyak T.M., Kołodyńska D. (2017). Metal ions removal using nano oxide Pyrolox™ material. Nanoscale Res. Lett..

[133] Kołodyńska D., Gęca M., Skwarek E., Goncharuk O. (2018). Titania-coated silica alone and modified by sodium alginate as sorbents for heavy metal ions. Nanoscale Res. Lett..

